# Dietary Interventions and Physical Activity as Crucial Factors in the Prevention and Treatment of Metabolic Dysfunction-Associated Steatotic Liver Disease

**DOI:** 10.3390/biomedicines13010217

**Published:** 2025-01-16

**Authors:** Paweł Rajewski, Jakub Cieściński, Piotr Rajewski, Szymon Suwała, Alicja Rajewska, Maciej Potasz

**Affiliations:** 1 Department of Internal and Infectious Diseases, Provincial Infectious Disease Hospital, 85-030 Bydgoszcz, Poland; 2 Faculty of Health Sciences, University of Health Sciences in Bydgoszcz, 85-067 Bydgoszcz, Poland; 3 Department of Radiology, Provincial Infectious Disease Hospital, 85-030 Bydgoszcz, Poland; jakub.ciescinski@gmail.com; 4 Department of Neurology, Collegium Medicum—Faculty of Medicine, Nicolaus Copernicus University in Toruń, 85-094 Bygoszcz, Poland; praj@poczta.onet.pl; 5 Department of Endocrinology and Diabetology, Collegium Medicum—Faculty of Medicine, Nicolaus Copernicus University in Toruń, 85-094 Bydgoszcz, Poland; szymon.suwala@abs.umk.pl; 6 University Clinical Hospital, 60-355 Poznań, Poland; alicja.p.rajewska@gmail.com (A.R.); potaszmaciej@gmail.com (M.P.)

**Keywords:** lifestyle, MASLD, non-pharmacological treatment, diet, physical activity, hepatic steatosis, cardiovascular disease, obesity, diabetes

## Abstract

Metabolic dysfunction-associated steatotic liver disease (MASLD) is the most common chronic liver disease worldwide and affects nearly 30% of the adult population and 10% of the pediatric population. It is estimated that this number will double by 2030. MASLD is one of the leading causes of hepatocellular carcinoma, cirrhosis, and liver transplantation, as well as a significant risk factor for cardiovascular disease and mortality. Due to the ever-increasing number of patients, the long-term asymptomatic course of the disease, serious complications, and lack of preventive programs, as well as insufficient awareness of the disease among patients and doctors themselves, MASLD is a growing interdisciplinary problem and a real challenge for modern medicine. The main cause of MASLD is an inappropriate lifestyle—inadequate nutrition and insufficient physical activity, which lead to various components of metabolic syndrome. Lifestyle changes—appropriate diet, weight reduction, and systematic physical activity—are also the basis for the prevention and treatment of MASLD. Hence, in recent years, so much importance has been attached to lifestyle medicine, to non-pharmacological treatment as prevention of lifestyle diseases. The narrative review presents possible therapeutic options for non-pharmacological management in the prevention and treatment of MASLD. The best documented and available diets used in MASLD were discussed, focusing on the benefits and drawbacks of the Mediterranean, high-protein, ketogenic, and intermittent fasting diets. In addition, the most recent recommendations regarding physical activity are summarized.

## 1. Introduction

Metabolic dysfunction-associated steatotic liver disease (MASLD) is the most common chronic liver disease worldwide, detected most often incidentally. It affects both adults and children, of both sexes, with a female predominance. In developed countries, MASLD may affect more than 30% of the adult population and 10% of the child population. However, this figure may be underestimated due to the long-standing asymptomatic course of the disease and the lack of screening programs, combined with low awareness of both patients and physicians regarding early detection of MASLD, especially among those at risk—excessive body weight, type 2 diabetes, dyslipidemia, or hypertension [1,2,3]. When diagnosed late, it can lead to the development of both hepatic and extrahepatic complications. It is one of the leading causes of hepatocellular carcinoma (HCC), cirrhosis, and liver transplantation in developed countries—the first cause of cirrhosis and liver transplantation in women in the USA and the second (after alcohol) in men. It is also a significant risk factor for the development of cardiovascular disease, which is the leading cause of death in this patient group (Table 1). Lack of awareness of the risk of developing the disease, the initial absence of clinical signs, or uncharacteristic symptoms means that MASLD is often diagnosed too late, at the stage of decompensated cirrhosis or after a first cardiovascular incident [4,5,6].

The main cause of MASLD is an inappropriate lifestyle—poor diet and lack of physical activity—which leads to the development of the metabolic disorders mentioned: overweight and obesity, pre-diabetes and diabetes, hypertension, and lipid disorders. These are typical components of the metabolic syndrome, i.e., a set of cardiovascular risk factors that, when present together, further increase this risk; hence, extrahepatic complications, i.e., cardiovascular incidents, are a frequent complication [1,2,5,7].

The diagnosis of the disease is based on the detection of hepatic steatosis by imaging in a patient with cardiometabolic factors and no other detectable cause of hepatic steatosis [1,3,5,6,7]. The most common imaging modalities used are classic ultrasound or elastography of the liver, e.g., FibroScan, which is used for the early detection of minimal steatosis (when 5% of hepatocytes are affected, compared to classic ultrasound methods, which detect 20–30% steatosis). For the diagnosis of MASLD, methods based on computed tomography or magnetic resonance imaging are used less frequently due to cost. Classical liver biopsy, on the other hand, is only used for clinically doubtful cases, overlap syndromes, or to differentiate with steatohepatitis. The treatment of patients with MASLD should be multidisciplinary and aimed at preventing and treating both hepatic and extrahepatic complications, i.e., reducing cardiovascular risk factors (cardiodiabetic factors) (Table 2). The most important aspect in the prevention and treatment of patients with MASLD is lifestyle change aimed at weight reduction [7,8,9,10,11,12,13].

**Table 1:** Complications of MASLD [6,7,11,13].

Hepatic Complications	Extrahepatic Complications
metabolic dysfunction-associated steatohepatitis (MASH) progression of fibrosis—F0 to F4—cirrhosis primary liver cancer liver failure and need for liver transplantation	arteriosclerosis cardiovascular diseases ischemic heart disease myocardial infarction TIA and ischemic stroke

**Table 2:** Pillars of the MASLD treatment [7,11,13].

Pillar I	Treatment of obesity with target weight reduction—optimally 10% of baseline weight within 6 msc: Lifestyle changes: diet, physical activity. Pharmacotherapy. Treatment by bariatric surgery.
Pillar II	Elimination of cardiometabolic risk factors, which are the main cause of premature mortality in patients with MASLD: Optimal treatment of diabetes, Optimal treatment of lipid disorders, Optimal treatment of hypertension.
Pillar III	Use in patients with MASH of drugs that have demonstrated in clinical trials the ability to reduce MASH and/or regress liver fibrosis: Pioglitazone, Vitamin E, GLP-1 analogs (semaglutide and liraglutide), GLP-1/GIP analogues (thirzepatide)

Pharmacological treatment is particularly justified in patients with histologically confirmed metabolic-associated steatohepatitis (MASH), advanced liver fibrosis (≥F2), and/or associated comorbidities.

In patients with MASH, the use of vitamin E at a dose of 800 IU/day can be considered. The rationale for using vitamin E lies in its antioxidant properties. Randomized controlled trials have shown that high doses of vitamin E may lead to biochemical normalization (ALT, AST) and reductions in steatosis, inflammation, and ballooning degeneration in non-diabetic patients with MASH. However, no improvement in fibrosis has been observed. Vitamin E is currently rarely used due to concerns about its long-term effects, such as an increased risk of prostate cancer in men over 50 years old and stroke. Vitamin E is not recommended for patients with diabetes or liver cirrhosis [5,8,11,13].

Pioglitazone, a thiazolidinedione derivative, is approved for the treatment of type 2 diabetes. At a dose of 30 mg/day in patients with MASH, it can lead to histological improvements in steatosis and inflammation. However, due to adverse effects, its use is now limited. It may cause fluid retention, exacerbate heart failure, and increase the risk of bladder cancer. Pioglitazone should not be used in patients with advanced liver dysfunction [5,8,11,13].

Glucagon-like peptide-1 (GLP-1) receptor agonists, such as liraglutide and semaglutide, as well as GLP-1 and glucose-dependent insulinotropic polypeptide (GIP) receptor agonists, such as tirzepatide, have shown promising effects in clinical trials. Studies investigating GLP-1 or GLP-1/GIP analogs have demonstrated beneficial effects of these drug classes on reducing liver steatosis and fibrosis, as well as lowering cardiovascular risk [3,5,8,10,11,13].

In addition, hepatoprotective—hepato-regenerative drugs such as ursodeoxycholic acid (UDCA), thymonacin, or soy phospholipids are very popular. These drugs should be considered to support the effects of lifestyle changes—diet, weight reduction, and physical activity [11,12,13].

**Figure 1:**
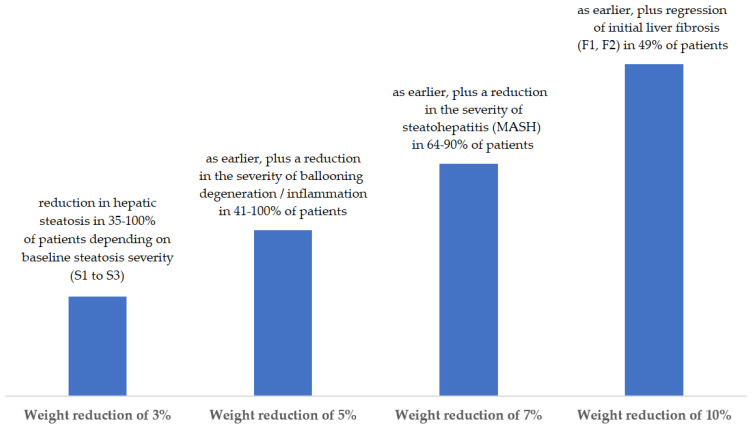
Effect of weight change on the course of MASLD [12].

The aim is not to lose weight quickly and significantly, but to lose weight in a way that will result in improved health and be sustained over the long term. Optimally, a slow weight loss of 0.5 kg to 1 kg per week is recommended, with a goal of reducing 5–10% of the initial body weight in 3–6 months (Figure 1). The reduced weight should then be maintained for a similar period, and, if indicated, weight should be reduced again by 5–10% in the following period [7,11,12].

The following presents possible therapeutic options for non-pharmacological management in dietary therapy for the prevention and treatment of MASLD. The most well-documented and accessible diets used in MASLD are discussed, focusing on the advantages and disadvantages of Mediterranean, high-protein, ketogenic diets, and intermittent fasting. The role of gut microbiota modification as a potential therapeutic option for MASLD is also addressed.

## 2. Dietary Recommendations in MASLD

A healthy diet, combined with regular exercise, is the mainstay of treatment for the vast majority of patients diagnosed with hepatic steatosis—this is in no way surprising, given that the presence and severity of hepatic steatosis are largely determined by excess energy intake, insulin resistance, and other factors regulating the supply and distribution of fatty acids, cholesterol, or phospholipids [14,15,16]. A fundamental problem, however, is the inconsistency in official dietary recommendations—while most scientific societies emphasize the importance of reducing excess body weight (usually using a hypocaloric diet with an energy deficit of 500–1000 kcal [3,17,18,19]), they rightly link the issue of hepatic steatosis to metabolic disorders, primarily obesity. There are discrepancies in specific recommendations—for example, the EASL-EASD-EASO and APASL guidelines recommend the exclusion of processed and high-fructose foods, whereas the AASLD and ESPEN guidelines do not [3,17,18,19]. Significant differences also apply to the supply of alcoholic beverages: EASL-EASD-EASO and AASLD allow moderate alcohol consumption in their recommendations, while the more recent ESPEN and APASL guidelines recommend complete abstinence [3,17,18,19]. Although official recommendations are sometimes inconsistent, there are nevertheless many individual reports in the scientific literature on different dietary interventions that may carry limited or global benefits for patients with features of hepatic steatosis; it is worth analyzing the most recurrent voices of the scientific community, which we will do later in this section, discussing the Mediterranean, high-protein, and ketogenic diets, as well as the intermittent fasting model.

### 2.1. The Mediterranean Diet

The Mediterranean diet is the traditional dietary approach of the Mediterranean people, characterized by a high proportion of low-processed foods and products: fresh fruits, vegetables, whole grains, legumes, nuts, pulses, fish, seafood, and extra virgin olive oil; fermented dairy products; with a low intake of animal fats and meat—a way of eating that contrasts strongly with the standard Western diet, rich in animal products, including red meat, refined cereals, or sweetened beverages [20,21,22,23,24,25,26]. Previous scientific reports emphasize the benefits of the Mediterranean diet in patients with metabolic diseases, made possible by its richness in antioxidants, monounsaturated fatty acids, fiber, well-digested animal protein, and polyphenols—the interplay of which reduces intrahepatic triglyceride accumulation, influences the expression of genes related to adipogenesis and adipocyte proliferation, sensitizes peripheral tissues to insulin (while regulating its secretion), and enhances the inflammatory response associated with adipose tissue activity [22]. Extra virgin olive oil, a primary fat source in the Mediterranean diet, comprises 55–83% monounsaturated fatty acids, predominantly oleic acid—this compound exhibits anti-inflammatory and immunomodulatory properties while also reducing DNA damage, CACT expression, hepatic paraoxonase activity, and hydrogen peroxide production [23]. Early cereals, referred to as “ancient wheat” (prevalent in the ancient Greek diet and endorsed by Galen of Pergamon as a fundamental food source), exert positive effects on insulin resistance, which underlies the pathophysiological mechanisms of liver steatosis and fibrosis [24]. Several independent scientific groups have shown that adherence to a Mediterranean diet in patients with previously diagnosed MASLD was associated with less steatosis (with reductions in intrahepatic fat volume reaching up to 39%), as well as a lower likelihood of developing features of hepatitis [3,19,20,21,22,25,26,27,28,29,30,31,32]. The group of Marin-Alejandre et al. showed (during a 6-month study that included 98 patients) that monounsaturated fatty acids, in particular, which are typical of the Mediterranean diet, may play a key role, improving the lipid profile and control of carbohydrate metabolism, reducing the phenomenon of insulin resistance, and having a beneficial effect on blood pressure, with a consequent reduction in intrahepatic fat volume and improvement in the clinical course of NAFLD [30]—these results were de facto confirmation of earlier studies, such as those by Bozzetto et al. [33]. Polyunsaturated fatty acids are no less important, especially maintaining an appropriate ratio of n-6 acids to n-3 acids—as it has been previously demonstrated (on animal models) that an appropriate meal composition in this regard (and especially an increased supply of α-linolenic acid relative to n-6 acids) improves peripheral insulin sensitivity and lowers cholesterol and triglyceride concentrations in cases of fructose-dependent insulin resistance burden [34]. Polyunsaturated n-3 fatty acids significantly reduce the activity and expression of the mitochondrial citrate carrier that catalyzes the efflux of citrate from the matrix towards the cytosol, which in turn leads to increased activity of acetyl-CoA required for de novo fatty acid and cholesterol biosynthesis [34]. In addition, n-3 fatty acids also up-regulate the expression of genes responsible for peroxisome proliferator-activated receptor alpha (PPARα) and sterol regulatory element-binding protein-1 (SREBP-1), which are responsible for fatty acid oxidation, lipogenesis, and glycolysis [35,36]. The anti-inflammatory effect of n-3 fatty acids cannot be underestimated either, related to the effect on suppression of TNFα and IL-6, which are typical cytokines responsible for the development and progression of MASH [37]. There are reports that the Mediterranean diet may be associated with a reduced risk of hepatocellular carcinoma and liver disease-related mortality [38,39]. Although there is a lack of randomized, high-quality studies collectively analyzing the Mediterranean diet, the EASL-EASD-EASO, ESPEN, and APASL guidelines list it as specifically recommended for patients with MASLD [3,17,18]. However, the Mediterranean diet is not free of drawbacks—one of the main challenges of its use is the potential difficulty of adapting this dietary model to individual patients’ needs (especially given financial constraints and the availability of some ingredients, such as fresh fruit, vegetables, and fish) [40]. It is also important not to overdo it—the Mediterranean diet, although healthy in principle, can also be hypercaloric, especially with an excessive supply of olive oil or nuts; people with hepatic steatosis should therefore, as always, be aware of the amount of calories consumed.

### 2.2. Protein-Rich Diets

Protein-rich diets are another type of diet discussed in terms of their potential beneficial effect on hepatic steatosis. The results of studies are inconsistent, on the one hand noting a significantly higher protein intake in patients with MASLD features [41,42], but there are also studies noting such a statistically and clinically significant correlation [43,44,45]—a solution to this controversy may be to look more closely at the quality and source of protein, as Rietman et al. noted (in a large cross-sectional study among a general Dutch adult population) that there is an inverse relationship between plant protein intake and MASLD features, while animal protein intake was associated with greater intrahepatic lipid accumulation [46]. Researchers from Taiwan have demonstrated that antioxidant-containing soy protein can improve liver function even at the MASLD stage—this is achieved by reducing plasma free fatty acid concentrations, decreasing CYP2E1 expression, increasing superoxide dismutase activity, and consequently reducing the action of lipid peroxidation products, including malondialdehyde and 4-hydroxy alkenes, among others. The beneficial effect of plant-derived proteins (including soy) on the regulation of the inflammatory response and the activity of the immune system by affecting TNFα concentrations is also not negligible [47]. High consumption of meat, especially processed meat, is associated with impaired diabetes susceptibility and, consequently, increased prevalence of specific features of the metabolic syndrome and its individual components, with a particular focus on obesity and type 2 diabetes and associated features of MASLD—Babio proved this during the high-quality 1-year PREDIMED trial, with usage of a 137-item validated food frequency questionnaire on a big cohort of 739 patients with high risk of cardiovascular diseases [48]. However, with this in mind, the benefits of a high-protein dietary model cannot be overlooked—Xu et al. demonstrated (although it must be admitted that it was based on a small group of 19 patients with morbid obesity) that a 30% protein diet was associated with a 42.6% decrease in intrahepatic fat concentrations, which was linked to beneficial effects on hepatic autophagy and reduced inflammatory response [49]. A prospective study by Markova et al. involving 37 subjects with NAFLD and type 2 diabetes, who were placed on an isocaloric diet rich in either animal or plant protein, demonstrated that, irrespective of the protein source, this diet resulted in a reduction in liver fat volume—furthermore, a reduction in the concentration of keratin-18 (an indicator of necroinflammation in the liver) was noted exclusively in the cohort of patients adhering to a plant protein-rich diet [50]. The EASL-EASD-EASO guidelines include a protein-rich diet as one of the potentially beneficial lifestyle change interventions in MASLD [17]. The ESPEN guidelines state that patients with features of obesity-related disease, hepatic steatosis, and comorbidities should follow a hypocaloric diet with an increase in target protein intake (2.0–2.5 g/kg body weight, as recommended by the American Society for Parenteral and Enteral Nutrition for critically ill obese patients [51]), which is expected to contribute to the reduction in fat mass and an increase in insulin resistance—however, interestingly, this recommendation does not have the full approval of the review board (71% agreement) [3]. Perhaps this is related to concerns about the negative impact of high-protein diets on the development of de novo chronic renal failure, as described by Ko et al.; however, it is worth noting that the concern is mainly with animal protein diets, characterized by high phosphate content [52]. To mitigate concerns regarding the onset of renal function disorders, it is advisable to utilize plant-based proteins (which are known to positively influence hepatic necroapoptosis and also exert beneficial effects on the kidneys because substituting one serving of red meat with a plant-based protein, such as legumes, was linked to a 31–62.4% reduction in the risk of chronic kidney disease [49]. For patients with pre-existing disorders, it is essential to limit sodium intake, ensure sufficient fiber consumption, and maintain body mass index within normative ranges [53].

### 2.3. Ketogenic Diets

For many years, the scientific community seems to have become increasingly interested in the therapeutic potential of ketogenic diets, mainly characterized by a low carbohydrate and high fat supply. Ketogenic diets may have a beneficial effect on patients with MASLD features by enhancing insulin sensitivity (which happens due to a reduction in the supply of simple carbohydrates, especially fructose, and a secondary reduction in body weight [53,54]), reducing hepatotoxic oxidative stress with a subsequent increase in mitochondrial efficiency [55,56] as well as the effect on the intestinal microbiota (patients with MASLD and MASH features are characterized by reduced activity of the bacteria Rikenellaceae, Ruminococcaceae, Faecalibacterium, Coprococcus, Anaerosporobacter, and Eubacterium, and the ketogenic diet leads to an increase in the abundance of precisely the short-chain fatty acid-producing bacteria beneficial for metabolism [57,58]). The ketogenic diet leads to a state of ketosis in which the body uses ketone bodies rather than glucose as the main energy source—this results in a reduction in insulin and insulin-like growth factor concentrations and an increase in fatty acid oxidation, which in sum modulates the inflammatory response and protects the liver from damage through lipid accumulation [59,60,61]. Rinaldi et al. on a group of 33 patients following a very low-calorie ketogenic diet demonstrated that it was effective in reducing hepatic steatosis as assessed by elastography (Fibroscan)—patients characterized by a baseline CAP suppression parameter of 266.6 ± 67 dB/m after 8 weeks obtained a decrease in this parameter to a level of 223 ± 64 dB/m, also obtaining an average reduction in BMI of 3 kg/m^2^ and in body fat mass of 7.5 kg [62]; similar effects with the same dietary model (consumption of 20–50 g of carbohydrates per day, 15–30 g of fat and protein at 1–1.4 g/kg of body weight) were also obtained by De Nucci et al. (on a bigger cohort of 87 patients) [63]. The group of Vilar-Gomez et al. showed that following a non-restrictive ketogenic diet (with carbohydrate intake <30 g/d, protein of 1.5 g/kg of body weight, and fat to satiety), compared to the standard dietary model proposed by the American Diabetes Association, was characterized in patients with MASLD on a diabetes background by greater success in weight loss, improvement in laboratory parameters (HbA1c, fasting insulinaemia, HOMA-IR index, aminotransferases, C-reactive protein), and in non-invasive indices of hepatic steatosis and fibrosis—these findings are significant as they are from a year-long longitudinal study with a substantial cohort of 349 patients [64]. An interesting comparison of hypocaloric diets was made by Crabtree et al.—assessed weight loss and liver fat reduction between study participants on a ketogenic diet with additional ketone supplementation (carbohydrate supply 40 g, protein 99 g, fat 143 g daily), a ketogenic diet with placebo (carbo supply 38 g, protein 100 g, fat 131 g daily) and a high-carbohydrate low-fat diet (with carbohydrate supply 259 g, protein 100 g and fat 51 g per day)—it was found that, although after 6 weeks, the best weight reduction was achieved in patients on the ketogenic diet, at the same time, the strongest reduction in liver fat was seen in patients on the low-fat diet (however, only in absolute terms, with no statistically significant differences noted between groups) [65]. A certain difficulty in the pooled analysis of ketogenic diets is that the dietary models based on this concept are not consistent—the most common differences are in the different proposed percentages or macronutrient weights, as well as the suggested pooled calories. Legitimate concerns regarding the use of a ketogenic diet include the possibility of nutritional deficiencies and an increase in LDL fraction cholesterol (albeit without maintaining the overall negative health atherogenic profile) [66]. To counteract deficiencies, supplementation of water-soluble vitamins (thiamin, riboflavin, niacin, vitamin B6, folic acid, biotin, and pantothenic acid in sugar-free formulations) and zinc, selenium, calcium, carnitine, and omega-3 fatty acids should be considered [67]. The only guidelines that include the low-carbohydrate ketogenic diet as one of the recommended dietary interventions (without going into the details of this dietary model) are those by EASL-EASD-EASO [17].

### 2.4. Intermittent Fasting Model of Diets

An interesting concept of dietary intervention that does not strictly focus on the composition of meals but rather on the timing of their intake is the intermittent fasting model. In practice, intermittent fasting has been used since the beginning of time, mainly for religious or cultural reasons [68,69]. The potential to stabilize the diurnal rhythm of hormone secretion (specifically insulin and cortisol [70,71]) is considered to be a factor promoting the efficacy of intermittent fasting in nutritional therapy for MASLD, regulation of the secretion of adipokines and inflammatory biomarkers derived from visceral adipose tissue (leptin, adiponectin, resistin, IL-6, and TNFα [72,73]), effects on the gut microbiota, or activation of autophagy with concomitant stimulation of growth hormone secretion [74,75,76]. Johari et al. demonstrated (in a randomized controlled trial with per-protocol and intention-to-treat analysis on 43 individuals with NAFLD) a positive effect of applied temporary caloric restriction on ALT levels and hepatic steatosis assessed using magnetic resonance imaging [77]—similar results in their study using liver elastography were obtained by the Australian group Feehan et al. (on a group of 34 patients during a 12-week trial) [78]. Dietary models based on time restrictions are characterized by a relatively high degree of acceptability—admittedly, there are known reports of sleep architecture disturbances occurring during the typical Ramadan fasting phase, but at the same time without cognitive and physical impairment in fasting subjects, and even with improvement in mood disturbances if previously present [77]. As with the ketogenic diet, due to the abundance of diverse protocols for this dietary model, it is difficult to generalize the results of the studies to date; despite this, an umbrella review of 11 meta-analyses and 130 RCTs by an international consortium of researchers demonstrated the health benefits of intermittent fasting: weight loss with a reduction in mainly body fat mass, improvement in lipid metabolism parameters, reduction in the severity of insulin resistance and inflammation, and a decrease in blood pressure [78].

It is also worth looking at attempts to compare intermittent fasting interventions with other dietary interventions. Unfortunately, the existing literature is relatively poor in comparative analyses of intermittent fasting interventions. A meta-analysis conducted by Guerrero et al. in 2021, encompassing 18 studies, failed to yield definitive conclusions about the benefits of this diet compared to other diets that employ continuous energy restriction [79]. In the same year, Holmer et al. conducted a comparison of intermittent calorie restriction and a low-carbohydrate, high-fat diet among 74 patients with NAFLD over a 12-week period—both interventions resulted in a significant reduction in liver steatosis, with no notable differences between them (however, an exception was observed in liver stiffness, which decreased in the 5:2 diet but not in the ketogenic diet intervention) [80]. Lee et al. in 2024 conducted a 12-week study involving 63 patients, revealing that intermittent fasting resulted in a more substantial reduction in liver fat content compared to the standard-of-care diet, despite an insignificant difference in body weight loss [81]. A definitive head-to-head comparison between time-restricted diets and other dietary approaches, such as the Mediterranean or protein-based diets, is lacking, as is a thorough global evaluation of all prevalent nutritional treatments for MASLD. Although it seems that the effect of intermittent fasting diets on liver function and possible structural abnormalities is beneficial and not solely reliant on weight loss, additional large-scale, high-quality research is required to establish definitive conclusions.

### 2.5. Summary About Dietary in MASLD

As in most cases, the key to appropriate nutritional therapy in hepatic steatosis remains its individualization and personalization [82]. In a logistic regression model by Perez-Diaz-del-Campo et al. (conducted during a 6-month trial where 87 patients with MASLD were randomly assigned to one of the three dietary arms), it was shown that a personalized diet (low-carbohydrate or Mediterranean) to give a reduction in hepatic steatosis must simply be characterized by a decrease in body weight and therefore be primarily hypocaloric [83].

Due to the diverse tastes and dietary preferences of each patient, it is challenging to identify a singular suitable nutritional model for everybody. Given the scarcity of high-quality data on restrictive diets, the most sensible choice for the treatment of hepatic steatosis seems to be an individualized hypocaloric diet based on healthy eating patterns, including regular meals eaten at appropriate times, using unprocessed or minimally processed foods, limiting easily digestible simple sugars and saturated fats, yet high in polyphenols and n-3 polyunsaturated fatty acids. The strongest evidence for the efficacy of nutritional treatment seems to be (as described above) the Mediterranean diet, which is additionally relatively easy to convert to individually tailored models according to the patient’s experience and preferences [3,17,18,84].

However, Table 3 provides an overview and characterization of the most significant studies on dietary treatments mentioned in this publication, allowing the reader to form their own conclusions.

### 2.6. Alternative Possibilities of Dietary Treatment in MASLD

Bacterial flora overflow is seen as a potential cause of fatty liver disease, prompting the scientific community to investigate the therapeutic effect of probiotics [85]. Previous reports indicate that suitably chosen probiotic treatments, particularly when combined with synbiotics, may confer advantageous effects in the adjunctive management of fatty liver disease and fibrosis [86,87,88,89]. These formulations predominantly consist of Bifidobacterium longum, Lactobacillus paracasei, Lactobacillus johnsonii, and Lactobacillus reuteri, among others, which are intended to mitigate insulin resistance, the adverse effects of dyslipidemia, and systemic inflammatory conditions [90,91]. However, further research is certainly necessary in this area because, as the authors of both meta-analyses noted, in most studies liver biopsy was not the gold standard for observing efficacy.

An intriguing subject increasingly explored in research is the potential application of natural products, such as Mastiha, a resinous exudate from the Pistacia lentiscus tree native to the Mediterranean region. This substance comprises a diverse array of phenolic compounds, phytosterols, arabino-galactans, proteins, and terpenes. Evidence suggests it possesses antioxidant properties, likely due to the inhibition of protein kinase, as well as anti-inflammatory effects resulting from the suppression of NF-kB [92]. Amerikanou et al. demonstrated in the MAST4HEALTH randomized, controlled trial that Mastiha supplementation ameliorates microbiota dysbiosis and lipid metabolite levels in patients with liver steatosis, with those suffering from advanced obesity experiencing greater reductions in liver fibrosis parameters [93].

Curcumin is a polyphenolic compound categorized as a curcuminoid and is a notable topic of investigation for MASLD. The origin of this substance is turmeric (Curcuma longa), a plant from the ginger family indigenous to Asia, particularly India, where it is predominantly utilized as a spice for its flavor, fragrance, and vibrant yellow hue. Researchers have consistently highlighted the antioxidant [94], anti-inflammatory [95], and potential anticancer effects of curcumin [96]. Kong et al. demonstrated that curcumin, owing to its antioxidant properties, mitigates the elevation of reactive oxygen species (ROS) and modulates autophagy—crucial in the pathogenesis of liver fibrosis—thereby reducing epithelial–mesenchymal transition and exhibiting antifibrotic effects [97]. The literature review conducted by Różański et al. indicates that curcumin supplementation may positively influence biochemical parameters associated with liver, kidney, and adipose tissue function, thereby affecting liver steatosis and fibrosis. However, the authors caution that the variability in individual study results precludes definitive recommendations regarding its therapeutic potential [98].

There are certainly more natural products that constitute an alternative or unconventional approach that may cooperate with dietary interventions—time and the results of further scientific studies will show which of these warrant attention [99].

The following presents possible therapeutic options for non-pharmacological management through physical activity in the prevention and treatment of MASLD. The importance of regular physical exercise in the prevention and treatment of MASLD is discussed and summarized.

## 3. Recommendations for Physical Activity in MASLD

Patients with MASLD, especially those with obesity, type 2 diabetes, hypertension, or a history of cardiovascular incidents, as well as elderly patients, should consult their doctor before deciding on regular physical activity, in addition to regular walking, in order to assess their clinical condition and any contraindications to exercise and to determine the frequency and intensity of exercise. Exercise should be introduced gradually, especially in people who have not been physically active before and at low intensity, thus avoiding overtraining and injury (Table 4).

Regular physical activity helps to improve fat metabolism and tissue sensitivity to insulin, as well as reducing insulin resistance and fat deposition in the liver [100].

A number of observational studies have shown that exercise reduces the incidence of MASLD. In an Italian cross-sectional study of 191 people, an inverse correlation was found between liver fat content and regular exercise [101]. In a Dutch study of 42,661 people, even lower levels of physical activity than the recommended minimum of 150 min per week were shown to have positive effects. The greatest results occur in diabetic and elderly patients [102]. In a cross-sectional study of 139,056 Koreans, spending more than 5 h in a sedentary position during the day was shown to increase the chances of MASLD being found on ultrasound [103]. Another Korean study found that people who exercised at least three times a week for at least 30 min for more than three months halved their risk of developing MASLD [104].

Physical activity can reduce the risk of developing MASLD by acting on multiple factors [6]. Aerobic exercise can reduce visceral adipose tissue and adipocyte size, which reduces the accumulation of free fatty acids in the liver [105]. During exercise, glucose uptake and storage as glycogen by muscle tissue increases. Regular exercise increases the uptake and oxidation of fatty acids by muscle [106]. Physical activity also affects the liver itself through multiple mechanisms—exercise is responsible for reducing oxidative stress, inflammation, and the fibrosis process, decreasing de novo lipogenesis, and increasing beta-oxidation of fatty acids occurring in the liver [107]. Exercise modulates the gut microbiota, increasing its diversity and changing the ratio of individual bacterial strains in favor of a phenotype less conducive to hepatic steatosis. They also improve the intestinal barrier and bile acid homeostasis [108].

A number of randomized controlled trials and meta-analyses have been conducted to assess the effect of aerobic exercise on the treatment of MASLD. Exercise-only interventions led to reductions in liver fat ranging from 2% to 50% [109]. In addition, exercise also had a moderate effect on lowering aminotransferase levels [110]. It has been shown that a 1% weight loss corresponds to a 1% decrease in liver fat [111].

A greater effect of physical activity on the reduction in hepatic steatosis was reported in those with a diagnosis of MASLD and a higher baseline BMI [110,111,112,113]. Studies indicate a relationship between fat reduction and total training time [110]. However, the optimal duration and intensity of exercise needed to reduce hepatic steatosis remain uncertain and require further research.

In a meta-analysis of 17 studies, it was shown that for each week of exercise, liver fat levels decreased by 0.27% [111]. Keating et al. conducted a study on the effects of varying exercise intensity on liver fat. The study was conducted on a group of 48 patients who were divided into four groups. The authors found no differences in visceral or hepatic fat reduction in patients exercising at different intensities and frequencies [114].

However, in a study involving 169 patients undergoing a 12-week training intervention, a greater reduction in liver fat was observed with high-intensity exercise compared to moderate intensity. The level was measured using the CAP parameter in liver elastography using the Fibroscan^®^ method (32% vs. 23%) [115].

In another meta-analysis of 16 clinical trials involving 706 subjects, it was shown that even physical exercise not accompanied by a change in diet can reduce liver fat [116].

The effect of resistance exercise, compared to aerobic exercise, on MASLD is less clear, and there is considerable heterogeneity in the findings. Studies suggest that aerobic exercise has a stronger effect on visceral fat reduction and regulation of glucose and lipid metabolism compared to resistance training [116].

Nevertheless, despite the lack of direct evidence of a beneficial effect of resistance training on hepatic steatosis, it has led to the maintenance of lean muscle mass during weight loss and improved muscle strength, muscle function, and insulin sensitivity, which argues for its addition to aerobic training in people with MASLD [116,117].

As Wu et al. showed in their pooled analysis, the combination of diet and exercise results in a weight loss of 1.1 kg greater than diet alone [118]. Analyses of fatty liver biopsy results, on the other hand, showed that the combination of a hypocaloric diet and the recommendation to walk 200 min per week was associated with significantly significant clinical benefits in terms of steatosis, fibrosis, and liver function [119]. Engaging in physical activity alone, even if not associated with dietary management and weight loss, is effective in reducing intrahepatic and peripheral triglyceride concentrations [106,120].

However, it is important that exercise becomes a regular part of patients’ behavior (the effects do not last longer than 12 months after cessation of regular exercise) and that it is not too strenuous (as this does not increase the effectiveness in reducing hepatosteatosis) [121,122]. Official guidelines for the management of MASLD are inconsistent, and the most precise guidance can be found in the EASL-EASD-EASO guidelines—they suggest that patients with MASLD should undertake moderate-intensity aerobic activity for 150–200 min per week (in 3–5 sessions) and at the same time exploit the benefits of resistance training.

Regular aerobic exercise such as brisk walking, Nordic walking, etc. has a beneficial effect on the remission of hepatic steatosis mainly due to the regulation of fatty acid oxidation (using adiponectin and AMP kinase), leptin, intrahepatic SREBP-1c levels and the action of antioxidant enzymes [123,124].

Resistance exercise, on the other hand, has been associated with improved health in patients, particularly those with carbohydrate metabolism disorders—although data on the effect of this type of exercise on hepatic fat accumulation are inconsistent, due to its beneficial effects on strength and endurance capacity (and therefore facilitation of aerobic exercise progression), this type of training should be considered beneficial for patients with MASLD [125].

In patients with MASLD, it is also worth paying attention to daily non-exercise physical activity, or what is known as NEAT, which stands for non-exercise activity thermogenesis, which is the energy spent on daily activities that are not formal training or exercise.

NEAT encompasses all movements and activities during the day, such as walking (e.g., to work, shopping, walking the dog, walking while on the phone), standing up and sitting down, housework (cleaning, cooking, laundry, washing up, gardening), involuntary movements such as toe tapping, fidgeting, or moving the legs while sitting, and occupational work that requires physical activity (e.g., standing, walking around the office).

People who are more active in their daily activities have been shown to burn more calories, which can support weight maintenance or reduction. Differences in NEAT can be as much as a few hundred calories per day between people with different lifestyles and, on a weekly, monthly, or yearly basis, make a significant difference in weight loss.

NEAT is particularly important for people who do not have time for regular training, as daily small activities can significantly increase their total energy expenditure [116,126,127,128,129].

Various mobile apps, fitness bands, and classic pedometers are interesting options for controlling quantitative exercise. The recommended number of steps per day for patients with obesity and MASLD is 10,000 steps. However, in the beginning, for those starting regular physical activity, this can be 4000–5000 steps per day, which should be gradually increased. On the other hand, the number of steps in seniors should be adjusted individually—mandated between 6000 and 8000 steps per day or as many as the senior’s health condition allows using the principle that every step counts. It is worthwhile using the aforementioned apps to enable notifications reminding of the need to move during the day [130,131,132,133].

Physical activity recommendations should be aimed at all patients with MASLD. When implementing physical activity, it is important to remember to tailor training on an individual basis, taking into account physical capacity, co-morbidity, and other potential factors hindering the initiation of regular physical activity, adherence to recommendations and patient cooperation. Factors cited in the literature include insufficient patient education about the benefits of physical activity and exercise technique itself, fatigue, lack of energy, pain, fear of falling and pain, as well as decreased motivation and willpower in those with anxiety and depressive disorders, which are often observed in patients with MASLD. In this group of patients, regular group exercises, bringing together patients with obesity and MASLD, which have a positive effect on motivation and maintaining the willpower of patients, and sports and rehabilitation holidays combined with nutritional education, therefore seem ideal. Any physical activity in patients with MASLD is extremely important, ranging from leisurely walks, cycling, jogging, Nordic walking, swimming in the pool, or tennis to team games such as volleyball, basketball, or resistance exercises and gym workouts. General recommendations recommend 150–300 min/week of moderate-intensity physical activity (3–6 MET) or 75–150 min/week of high-intensity (>6 MET) [134,135,136,137,138,139].

**Table 4:** Recommendations for physical activity in MASLD [11,134,135,136,137,138,139].

Category	Recommendations
Type of physical activity	Aerobic exercises: running, brisk walking, cycling, and swimming. These are particularly effective in reducing liver fat.
	Resistance training: weightlifting or resistance exercises help build muscle mass and improve metabolism.
Frequency	At least 150–300 min of moderate activity per week (e.g., brisk walking)
	Alternatively: 75–150 min of vigorous activity per week (e.g., running and interval training).
Intensity	Moderate intensity (3–6 MET): accelerated heart rate, but still able to maintain a conversation.
	High intensity (>6 MET): difficulty talking and increased breathing effort.
Continuity and regularity	Exercise should be performed regularly, ideally in distributed sessions, e.g., 30 min a day for 5 days a week.
	Even short periods of activity (e.g., 10-min sessions) can be beneficial if performed regularly.
Individual recommendations	Activity should be tailored to the patient’s health status, age, fitness level, and coexisting conditions (e.g., diabetes and hypertension).

## 4. Summary

Metabolic dysfunction-associated steatotic liver disease is now a real challenge for modern medicine, as are other lifestyle-dependent diseases dependent on excessive body weight. It is the most common liver disease in the world, and its prevalence is increasing year on year in both adults and children, which is very worrying. This disease has led to a change in the epidemiology of causes of cirrhosis and causes of liver transplantation in developed countries in recent years, where MASLD is beginning to dominate. This is mainly related to the growing epidemic of obesity in all age groups, associated with poor eating patterns and lack of regular physical activity.

The main treatment for patients with MASLD is lifestyle change, targeting dietary treatment and increased exercise to translate into weight loss and improved cardiometabolic parameters. On the one hand, this is the simplest, easiest, and cheapest recommendation with a proven impact on the course of MASLD, but it is also the most difficult to implement and, at the same time, to maintain in the long term by the patient, which usually ends in repeated effects—yo-yo (recurrence of obesity), further adversely affecting the course of hepatic steatosis and associated diseases. In dietary management, it is recommended to avoid processed products, especially those rich in saturated animal fats, rich in trans isomers, and foods containing fructose (Table 3). It is worth recommending alcohol abstinence (it is currently recognized that there is no safe dose of alcohol for people with liver disease). Regular drinking of coffee in the number of ≥3 cups a day and dietary modification of the intestinal microbiome—using a diet containing prebiotics (fiber, e.g., from vegetables and fruits), probiotics (fermented products, e.g., yogurts, kefirs, and pickles)—may have a beneficial effect on the course of MASLD, avoiding unnecessary antibiotic therapy or proton pump inhibitor (PPI) use. In overweight or obese patients, it is recommended to reduce caloric intake by 500–1000 kcal/d in order to gradually reduce body weight by 0.5–1 kg per week, optimally 10% of the initial weight within 6 months. Regular physical activity is recommended, at least to the extent recommended by the World Health Organization or, in the event of existing limitations, adapted to the current and individual capabilities of the patient (Table 4) [139,140].

Therefore, it seems necessary to educate the public from an early age about lifestyle medicine as a long-term prevention of MASLD, obesity, and cardiovascular disease. It is important to pay attention to and continually encourage regular physical activity and adherence to healthy eating principles.

## Figures and Tables

**Table 3:** Summary of the most significant clinical trials focused on various dietary interventions.

Study	Duration of Assessment	Patients Analyzed	The Most Important Outcomes Linked to Liver Health
The Mediterranean diet			
Bozzetto et al. (2012) [33]	8 weeks	36 patients with type 2 diabetes, who were overweight or obese	Improvement in percentages of liver fat, HbA1c, and activity of plasma AST.
Marin-Alejandre et al. (2019) [30]	6 months	76 patients with overweight or obesity with confirmed liver steatosis	Improvement in body composition, diastolic and systolic blood pressure, and all assessed biochemical parameters (including lipid profile, fasting glucose, HOMA-IR, C-reactive protein, leptin, and adiponectin).
Protein-rich diet			
Markova et al. (2017) [50]	6 weeks	37 patients with NAFLD and type 2 diabetes	Reduction in intrahepatic fat (by 48% during a diet rich in animal protein and 35.7% during a diet rich in plant protein), decrease in liver enzymes in serum (without differences between groups), and decreased markers on necroptosis in the liver in a group of patients on plant proteins (but not on animal proteins).
Xu et al. (2020) [49]	3 weeks	19 patients with morbid obesity	Improvement of intrahepatic lipid levels only in the group with high intake of proteins, lower expression of fat uptake and lipid biosynthesis genes, and lower activity of inflammation; no changes in hepatic mitochondrial activity and expression of genes responsible for oxidation.
Ketogenic diet			
Luukkonen et al. (2020) [56]	6 days	10 patients with NAFLD and overweight or obesity	Decrease in liver fat volume by 31%, without a simultaneous noticeable change in liver fibrosis. Decrease in GGTP and ALP concentrations, without changes in ALT and AST (with a simultaneous decrease of 34% in the de Rittis index). Improvement in triglyceride concentration, without affecting other lipid profile parameters. Improvement in insulin sensitivity.
Rinaldi et al. (2023) [62]	8 weeks	33 patients with NAFLD and overweight or obesity	Decrease in hepatosteatosis in elastography exam by 17–20%, without significant influence on liver stiffness (fibrosis). Improvement in weight loss, waist circumference, and reduction in blood pressure, all assessed parameters of sugar and fat metabolism and some indicators of liver function (ALT, GGTP, without influence on AST concentration).
De Nucci et al. (2023) [63]	8 weeks	87 patients with NAFLD and overweight or obesity	Decrease in hepatosteatosis assessed by elastography by 20%, borderline significant decrease in liver fibrosis parameters (−4%). Improvement in weight loss and waist circumference, blood pressure, all tested parameters of sugar and fat metabolism, all liver function parameters except AST, reduction in platelet count (by 9%) without affecting other peripheral blood morphology parameters, without differences in C-reactive protein concentration.
Intermittent fasting diet			
Johari et al. (2019) [77]	8 weeks	33 patients with NAFLD	Reduction in steatosis (by 26%) and liver fibrosis (by 15%). Improvement in weight loss, all liver function indices, and fasting glucose, but no effect on lipid parameters.
Holmer et al. (2021) [80]	12 weeks	74 patients with NAFLD	Reduction in hepatosteatosis in both the group of patients undergoing intermittent fasting (5:2) and the ketogenic diet (without significant difference between them), reduction in hepatofibrosis and LDL-C concentration is visible only in the group of patients on the 5:2 diet.
Lee et al. (2024) [81]	12 weeks	63 patients with steatotic liver disease	Greater reduction in liver fat content compared to the standard-of-care diet (72.2% vs. 44.4%) with no noticeable difference in body weight reduction between the above groups.
